# A New *In Vivo* Screening Paradigm to Accelerate Antimalarial Drug Discovery

**DOI:** 10.1371/journal.pone.0066967

**Published:** 2013-06-25

**Authors:** María Belén Jiménez-Díaz, Sara Viera, Javier Ibáñez, Teresa Mulet, Noemí Magán-Marchal, Helen Garuti, Vanessa Gómez, Lorena Cortés-Gil, Antonio Martínez, Santiago Ferrer, María Teresa Fraile, Félix Calderón, Esther Fernández, Leonard D. Shultz, Didier Leroy, David M. Wilson, José Francisco García-Bustos, Francisco Javier Gamo, Iñigo Angulo-Barturen

**Affiliations:** 1 Tres Cantos Medicines Development Campus, GlaxoSmithKline, Tres Cantos, Spain; 2 The Jackson Laboratory, Bar Harbor, Maine, United States of America; 3 Drug Discovery and Technology, Medicines for Malaria Venture, Geneva, Switzerland; Université Pierre et Marie Curie, France

## Abstract

The emergence of resistance to available antimalarials requires the urgent development of new medicines. The recent disclosure of several thousand compounds active in vitro against the erythrocyte stage of *Plasmodium falciparum* has been a major breakthrough, though converting these hits into new medicines challenges current strategies. A new in vivo screening concept was evaluated as a strategy to increase the speed and efficiency of drug discovery projects in malaria. The new in vivo screening concept was developed based on human disease parameters, i.e. parasitemia in the peripheral blood of patients on hospital admission and parasite reduction ratio (PRR), which were allometrically down-scaled into *P. berghei*-infected mice. Mice with an initial parasitemia (P_0_) of 1.5% were treated orally for two consecutive days and parasitemia measured 24 h after the second dose. The assay was optimized for detection of compounds able to stop parasite replication (PRR = 1) or induce parasite clearance (PRR >1) with statistical power >99% using only two mice per experimental group. In the *P. berghei* in vivo screening assay, the PRR of a set of eleven antimalarials with different mechanisms of action correlated with human-equivalent data. Subsequently, 590 compounds from the Tres Cantos Antimalarial Set with activity in vitro against *P. falciparum* were tested at 50 mg/kg (orally) in an assay format that allowed the evaluation of hundreds of compounds per month. The rate of compounds with detectable efficacy was 11.2% and about one third of active compounds showed in vivo efficacy comparable with the most potent antimalarials used clinically. High-throughput, high-content in vivo screening could rapidly select new compounds, dramatically speeding up the discovery of new antimalarial medicines. A global multilateral collaborative project aimed at screening the significant chemical diversity within the antimalarial in vitro hits described in the literature is a feasible task.

## Introduction

Malaria is a parasitic disease caused in humans by protozoa of the genus *Plasmodium* that invade and destroy red blood cells during their asexual multiplication. The disease continues to be a major burden to public health and economic development globally with an estimated 217 million malaria cases every year, resulting in about 0.7 million deaths [1]. However, the actual incidence of malaria is probably underestimated in some important endemic areas [2].

Antimalarial drugs remain the mainstay for malaria treatment and control [3]–[5]. Artemisinin-based combination therapy (ACT) is recommended as first-line treatment for uncomplicated *P. falciparum* malaria [6], and its implementation has contributed significantly to reducing the malaria burden in many endemic countries and countering resistance to key antimalarial medicines [1], [6]. Yet, the recent reports of artemisinin resistance in the Cambodia–Thai border [7]–[9], highlight the need for the continued development of new medicines [6], [10].

The disclosure of large sets of compounds active in vitro against the erythrocyte stage of *P. falciparum* [11]–[16] is a major breakthrough that has dramatically changed the landscape of drug discovery in malaria. These compound sets provide thousands of potential starting points for drug development using novel chemical scaffolds, thus minimizing the probability of developing compounds with cross resistance against current antimalarials. However, only a very small fraction of the compound sets are likely to produce compounds with the balanced properties of a medicine, i.e. non-toxic, orally bioavailable, lacking drug–drug interactions and efficacious against *Plasmodium* spp. [4].

The estimated time to develop a drug from discovery to commercialization is ten to fifteen years [17]. Most projects start with the *a priori* selection of promising drug-like structures according to criteria based on structural characteristics, in vitro anti-parasitic activity, and in silico and/or in vitro absorption, distribution, metabolism and toxicity (ADMET) properties. These ADMET properties are usually optimized in an iterative process until one or several compounds are tested in rodent models of malaria using standard assays to demonstrate efficacy in vivo. Unfortunately, both the capacity of the standard approach to predict efficacy in vivo and the probability that a specific drug discovery project reaches the clinical phases are very low [18], [19]. Consequently, there is a high risk of sustained investment of resources in projects doomed to failure.

As an alternative, in vivo efficacy studies could be performed before compound optimization. This would ensure that effective compounds belong to families with an acceptable balance between ADMET properties and antimalarial activity in vivo. Optimization would, therefore, be streamlined and the investment of resources between the initiation and potential failure of the project minimized. In addition, this approach allows better prediction of the performance of a compound family in humans, thus reducing the risk of attrition in clinical development from lack of efficacy [20].

Most antimalarial medicines were identified through in vivo screening in avian or murine models [21]–[24]. Currently, the Thompson survival assay and the Peters’ 4-day test are the most widely used efficacy assays in malaria [25], [22]. Although valuable as investigative tools, neither of these tests is amenable to high-throughput screening. Thus, the Thompson survival assay requires relatively large numbers of animals and/or long observation times whereas the Peters’ 4-day test is not robust for error detection in large in vivo screens because the concentration of parasite is below the detection limit of microscopy and flow cytometry at treatment starting point. Importantly, neither test evaluates efficacy parameters that are directly relevant to malaria in humans. Given the large number of in vitro hits that need to be evaluated, the feasibility of a high-throughput, high-content, in vivo assay requires examination. There are three main criteria for the feasibility such an assay [26], it should: a) enable effective prioritization of compounds according to their predicted efficacy in humans, b) be robust while minimizing the number of animals per compound tested, and c) detect a reasonably high percentage of efficacious compounds in the set of antimalarial hits.

In this work, the feasibility of an in vivo screening approach is assessed as a strategy to rapidly identify starting points for drug discovery projects. The screening assay used a *P. berghei* murine model of malaria infection based on parameters of human disease, i.e. parasitemia in the peripheral blood of patients at the point of hospital admission and the parasite reduction ratio (PRR), defined as the ratio of the baseline parasite count to that following treatment. Of note, only two animals per experimental group were required. The assay was validated against standard antimalarials *versus* the Peter’s 4-day test and compared to human-equivalent data. The assay was used to investigate a sub-set of 590 compounds from the Tres Cantos Antimalarial Set (TCAMS) [13]. Around 11% of the compounds tested were found to be efficacious in vivo, of which about 25% were as efficacious as potent marketed antimalarials. The methods described provide a feasible strategy for high-throughput, high-content, in vivo screening. Thus, drug discovery resources can be focused on compounds with the highest likelihood of delivering new medicines against malaria.

## Materials and Methods

### Ethics Statement

Animal experiments were performed at the AAALAC-accredited GlaxoSmithKline Laboratory Animal Science facility in Tres Cantos (Madrid, Spain). All the experiments were approved by the GlaxoSmithKline Diseases of the Developing World Group Ethical Committee. The animal research complied with Spanish and European Union legislation on animal research and GlaxoSmithKline policy on the care and use of animals. Experimental and control animals infected with *P. berghei* were euthanized at the end of the assay (day 4 after infection), before developing severe malaria and all efforts were made to minimize suffering.

### Compounds and Reagents

Chloroquine diphosphate, quinine, pyrimethamine, mefloquine hydrochloride, amodiaquine dihydrochloride, pentamidine, azithromycin, doxicycline hydrochloride, primaquine biphosphate, dihydroartemisinin, sulfadoxin, methylcellulose, hydroxypropylethyl cellulose, hydroxipropil-β-cyclodextrine, benzyl alcohol and Tween-80 were obtained from Sigma-Aldrich (St Louis, MO). Artesunate was obtained from AAPIN Chemicals Ltd. (Abingdon, UK). Atovaquone, proguanil and GSK932121 were prepared at GlaxoSmithKline. Piperaquine phosphate hydrate was purchased from AK Scientific (Union City, CA) as a suspension in water 1% (FLUKA, Seelze, Germany).

For the dose–response experiments to validate the in vivo assay, antimalarials were prepared in different vehicles to maximize bioavailability as follows: saline (amodiaquine, chloroquine, doxicycline, quinine); saline, 0.2% ethanol, 0.02% acetic acid (azithromycin); water (piperaquine); water, 1% methylcellulose (atovaquone, GSK932121); water, 20% hydroxipropil-β-cyclodextrine (artesunate); water, 0.2% methylcellulose, 0.4% Tween-80 (mefloquine); water, 0.5% hydroxypropylethyl cellulose, 0.4% Tween-80, 0.5% benzyl alcohol (pyrimethamine); dissolved in 30% ethanol, 70% Tween-80 and then diluted 1∶10 with water (pentamidine). The antimalarials used as quality control during in vivo screening of the TCAMS sub-set were prepared as suspensions or solutions in water, 5% DMSO, 20% Captisol®.

### Parasites

Uncloned *P. berghei* ANKA was donated by Dr E. Dei-Cas and Dr L. Delhaes from the Institut Pasteur (Lille, France) [27]. Parasites were maintained frozen at −150°C. For each individual assay, an aliquot was thawed and injected intraperitoneally into three mice. Donor infected mice were produced after three in vivo passages, euthanized with CO_2_ and infected blood obtained by cardiac puncture.

### Mice

Experimental and control animals were specific pathogen-free 8–12-week-old females, body weight range 20–22 g. CD1 Swiss (Hsd:ICR) mice were obtained from Harlan Interfauna (Iberica, Spain) and immunodeficient NSG (NOD.Cg*-Prkdc^scid^ Il2rg^tm1Wjl^*/Sz) mice from Charles River Laboratories (L’Arbresle, France under license of The Jackson Laboratory, Bar Harbor, Maine, USA). Up to five animals were accommodated in Tecniplast® type IV cages with autoclaved dust-free corncob bedding (Panlab, Barcelona, Spain). Facilities were kept under a twelve hours light/dark period at a room temperature of 22±2°C and 40–70% relative humidity and air-conditioned with twenty air changes per hour. Filtered tap water and a γ-irradiated pelleted diet were provided *ad libitum*.

### Flow Cytometry

Parasitemia in peripheral blood of mice was measured as described previously [27]. Briefly, blood samples (2 µl) from the lateral tail vein of mice were collected into 0.2 ml of Dulbecco’s phosphate-buffered saline (DPBS), 0.025% (vol/vol) glutaraldehyde, 1 mM ethylenediaminetetraacetic acid (EDTA), pH 7.2, on V-bottomed 96-well plates and fixed at 4°C in the dark for at least 24 h and for up to 3 days. Suspensions of fixed cells (30 µl) were passed onto another clean V-bottomed 96-well plate, washed with DPBS at room temperature and re-suspended in 0.2 ml of 0.25% (vol/vol) Triton X-100 in DPBS for 5 min for permeabilization. After centrifugation, cells were re-suspended in 0.1 ml of DPBS containing 1 mg/ml RNAse A, and incubated for 30 min at room temperature in the dark. Finally, cells were stained by adding 0.1 ml of YOYO-1 (Molecular Probes, Leiden, The Netherlands) and 0.5 µM in DPBS to each well and incubating for 30 min at room temperature in the dark.

Samples were acquired in a FACScalibur flow cytometer (Becton Dickinson, San Jose, CA). Erythrocytes and leukocytes were gated in logarithmic forward/side dot plots and fluorescent emission was collected in photomultipliers through 530/30 (FL-1) or 585/42 (FL-2) band-pass filters. Compensation of YOYO-1 emission in FL-2 was established empirically by comparison of blood samples from uninfected and *P. berghei*-infected CD1 mice. A total of 10^5^ events were acquired in samples with parasitemia greater than 0.1% and 10^6^ for parasitemias below that percentage. Between samples, a tube containing PBS was acquired to minimize carry-over. The limit of quantification was 0.06% parasitemia. Leukocytes and cellular aggregates were excluded. Sample analysis used CellQuest Pro 5.2.1 (Becton Dickinson).

### Evaluation of In Vivo Antimalarial Therapeutic Efficacy

At day 0, CD1 mice were infected intravenously with 10^7^ infected erythrocytes (IE), obtained from *P. berghei*-infected donor mice, suspended in 0.2 ml of saline. Animals were separated randomly into two mice per group. At day 2 after infection, tail blood samples were taken for determination of parasitemia before oral administration of test compounds at a volume of 20 ml/kg body weight. Artesunate was administered at 3, 6, 12, 25, 50, 100, and 200 mg/kg; piperaquine at 0.2, 1, 3, 15, 50, and 200 mg/kg; amodiaquine at 0.5, 2, 25, 50, and 100; chloroquine at 0.5, 2, 10, 50, 100, and 200 mg/kg; pyrimethamine at 0.02, 0.1, 0.5, 2, 10, and 50 mg/kg; atovaquone at 0.02, 0.1, 0.5, 2, 10, and 30 mg/kg; GSK932121 at 0.1, 0.4, 2, 10, 50, and 100 mg/kg; mefloquine at 0.2, 1, 3, 10, 30, and 90 mg/kg; quinine at 10, 25, 75, 200, and 300 mg/kg; doxicycline at 5, 15, 30, 75, 150, and 300 mg/kg; azithromycin at 0.7, 3, 5, 12, 50, and 200 mg/kg; and pentamidine at 0.4, 2, 10, 40, and 70 mg/kg. Each group of mice received the first dose of test compound on day 2 and a second dose after 24 h. Tail blood samples for determination of parasitemia were taken on day 4, i.e. 24 h after the second treatment dose. Vehicle-treated *P. berghei*-infected controls (n = 2) were included with each group. Atovaquone (0.2 mg/kg and 1 mg/kg) was used in quality control assays.

The Peters’ 4-day test with minor modifications was used for validation purposes [22]. Female CD1 mice were infected intravenously with 10^7^ IE, obtained from *P. berghei*-infected donor mice, suspended in 0.2 ml sterile saline. Treatment by oral gavage (20 ml/kg of body weight) with standard antimalarial drugs or corresponding vehicles commenced 1 h post infection and then every 24 h for four consecutive days. Parasitemia was measured by flow cytometry in 2 µl tail blood samples taken 24 h after the last dose administered. Amodiaquine was included as the quality control for each in vivo assay.

In both assays, compound therapeutic efficacy was expressed as the effective dose (mg of product per kg of mouse body weight) that reduced parasitemia by 90% with respect to the vehicle-treated group (ED_90_).

### Statistical Analysis

Normality of the distributions of the variables assessed in experiments was analyzed using the D’Agostino–Pearson normality test. Comparison of the mean of each experimental group was analyzed by Student’s t test or one factor ANOVA followed by Dunnett’s post test. Homogeneity of variances was assessed by Levene’s test. Data variability was expressed as standard deviation. Analysis was performed using GraphPad Prism 5.0 for Windows (GraphPad Software, San Diego, CA). Probability values >0.05 were considered not significant.

## Results

### Design and Optimization of an In Vivo Screening Assay

The parasitemia in peripheral blood of patients on hospital admission was used as the target for parasitemia established in *P. berghei*-infected mice at the start of drug treatment (P_0_). To calculate the human-equivalent parasitemia in mice at P_0_, the total number of infected erythrocytes divided by the average human total body weight in grams (parasite density) was calculated and extrapolated to mice. The geometric mean of the total parasite burden in patients at treatment initiation is approximately 5×10^11^ parasites [28]. For a 70 kg adult, the log_10_ of the parasite density is about 9.85. In the mouse, assuming a body weight of 0.022 kg, 1.5 ml blood volume, 7×10^9^ erythrocytes/ml and a small percentage of sequestered parasites compared to the circulating pool, P_0_ was set at 1.5% parasitized erythrocytes.

**Figure 1:**
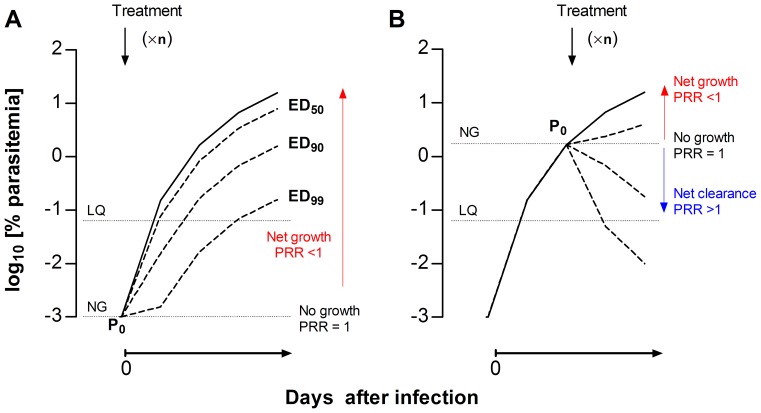
Concept of PNSA in vivo assays. Comparison of the theoretical growth curves of *Plasmodium berghei* upon intravenous infection at day 0 under (A) a Peters’ 4-day test-type or (B) the PNSA assay format for the evaluation of the antimalarial efficacy of drugs. The solid curves represent the growth of parasites treated with vehicle. The dotted lines represent the growth of parasites under arbitrary treatments (×n, denotes arbitrary number of drug dosages) leading to ED_50_, ED_90_ and ED_99_, respectively. The parasitemia that marks the limit between net growth and net clearance of the parasite circulating in peripheral blood is denoted as the NG line. The limit of quantification of parasitemia is denoted as the LQ line. PRR is the parasite reduction ratio, i.e. the ratio of the baseline parasite count to that following treatment.

The in vivo assay was designed as a P_0_-normalized screening assay (PNSA). In contrast with the Peters’ 4-day test (Figure 1A), drug treatment starts when mice have patent parasitemia (P_0_) (Figure 1B). This type of assay allows, a) visualization of the effects of drugs on parasites, and b) a clear cut off (P_0_, i.e. parasitemia when drug treatment starts) between parasite net growth in blood, if parasitemia increases with respect to P_0_ over time, or net clearance, if parasitemia decreases with respect to P_0_. Thus, the potency of a compound is expressed as the ratio of P_0_ versus the parasitemia at the end of therapy (Figure 1B), which is equivalent to PRR. The effective dose (ED) of a compound that maintains parasitemia at the end of the assay equal to P_0_ is the lowest limit of drug exposure required to prevent parasite growth in vivo (PRR = 1). If there is a net clearance of parasites, then the parasitemia at the end of treatment is <P_0_ and the PRR is >1 (Figure 1B).

**Figure 2:**
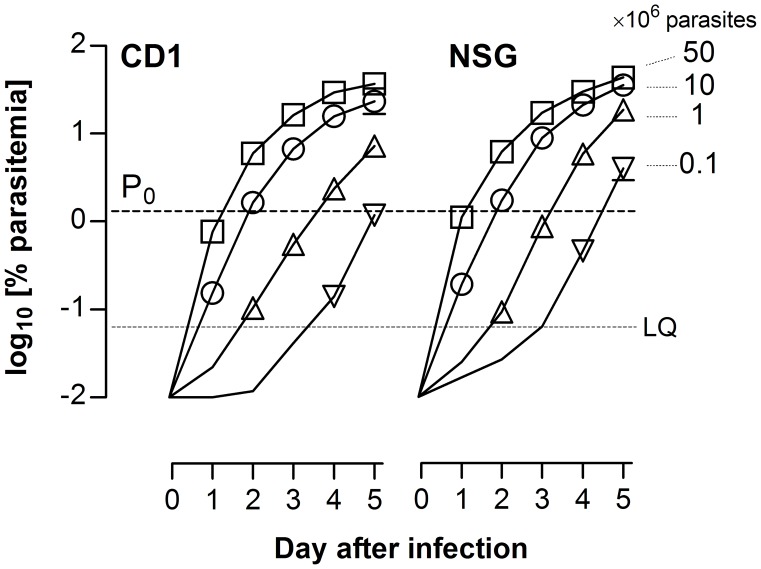
Selection of the infective dose for the*Plasmodium berghei* ED_90_-normalized in vivo assay. Growth kinetics of *P. berghei* following intravenous infection of (A) immunocompetent CD1 or (B) immunodeficient NSG mice is shown. The plots show the parasitemia in peripheral blood of female mice infected with 0.1×10^6^, 1×10^6^, 10×10^6^, and 50×10^6^ infected erythrocytes. The dashed line (P_0_) indicates the target human-equivalent parasitemia. Data are the mean ± standard deviation of n = 4 mice/group. Error bars are shown only if they are bigger than symbols.

*Plasmodium berghei* growth kinetics in CD1 mice defined the optimal assay duration. The inoculum size was used to establish the day of drug treatment initiation and the end of the assay in order to allow assessment of drug efficacy after treatment over a minimum of two parasite cycles. As shown in Figure 2, P_0_ ≈ 1.5% was achieved at days 1, 2 and 3 after infection with 50×10^6^, 10×10^6^, and 1×10^6^ IE, respectively. An initial inoculum of 10×10^6^ IE was selected to minimize the number of parasite-donor mice and to reduce assay duration. Consequently, day 2 was selected as start of treatment and day 4 as the end of the assay. This defines a period of exponential growth of *P. berghei* that is not dependent on the activity of the adaptive immune system, as similar growth kinetics were obtained in immunodeficient NSG mice (Figure 2).

The dynamic range can be used to define the most sensitive estimator of the ED. The dynamic range of the assay is defined as the difference between the limit of quantification (LQ) of the technique used to measure parasitemia and the parasitemia in vehicle-treated mice at the end of the assay (Figure 1B). In the specific implementation of the *P. berghei* screening assay presented in this paper, the LQ of the YOYO-1_530/585_ flow cytometry method was 0.06% [27], and mean maximum parasitemia in vehicle-treated mice at day 4 was 14.2±4.6% (n = 36 mice). Thus, P_0_ was nearly halfway in the dynamic range expressed in log_10_ scale. The potency of a compound in the assay is measured as the ED that reduces the log_10_ [parasitemia at day 4] to log_10_ [P_0_]. As P_0_ is about 90% of the maximum growth of *P. berghei* at day 4 after infection, the ED_90_ can be used as a reliable and sensitive parameter of potency in the screening assay (denoted as the ED_90_-normalized assay).

The ED_90_-normalized assay was designed to minimize animal use, but with high statistical power (>90%). The variable log_10_ [parasitemia at day 4] in mice infected with 10×10^6^ IE fitted a normal distribution of 1.14±0.14 (D’Agostino–Pearson normality test p* = *0.26) in an observational study of n = 40 mice pooled from 10 experiments. Using n = 2 mice per experimental group, to mitigate the risk of death for reasons unrelated to drug toxicity, the power of the assay for ED_90_ estimation was >99.9% (Type-II error β <0.1) for a 95% of confidence level (Type-I error α = 0.05) whereas the power to detect reductions in parasitemia of 50% was 85%.

In conclusion, tailoring the mouse model to parameters of human malaria by allometric down-scaling of parasitemia at treatment initiation leads to powerful and efficient experimental designs.

### Validation of the PNSA Screening Assay

The ED_90_-normalized assay against *P. berghei* ANKA was validated using a set of known antimalarial drugs with different mechanisms of action: artesunate, chloroquine, piperaquine, amodiaquine, pyrimethamine, atovaquone, mefloquine, quinine, azithromycin, doxicycline, the pyridone GSK932121 that inhibits *Plasmodium* spp. cytochrome *bc_1_*, and pentamidine, which is inactive against *P. berghei* in vivo [29].

**Figure 3:**
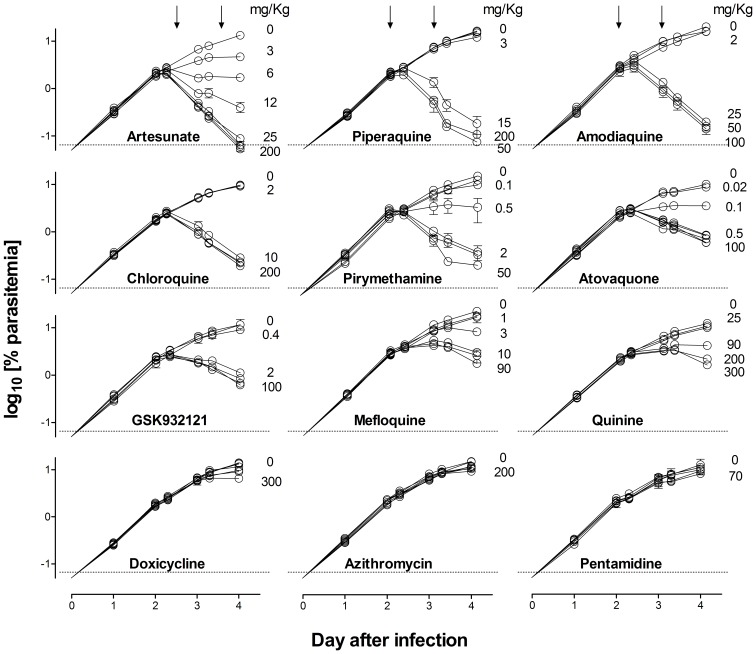
*Plasmodium berghei* clearance upon antimalarial treatment. The plots show the kinetics of parasitemia in peripheral blood of CD1 female mice infected with 10×10^6^ infected erythrocytes at day 0 and treated at days 2 and 3 (downward arrows) with a set of antimalarial drugs used for validation of the *P. berghei* ED_90_-normalized in vivo assay. For clarity, only selected doses are explicitly indicated in the plot. Data are the mean ± standard deviation of n = 3 mice/group. Error bars are shown only if they are bigger than symbols.

In the ED_90_-normalized assay, all the antimalarials tested except doxicycline, azithromycin and pentamidine showed a clear dose–dependent inhibition of parasitemia (Figure 3). Interestingly, for the antimalarials that were effective in this assay, there were obvious differences in the parasite clearance rate between compounds, except for atovaquone and GSK932121, which share a common mechanism of action (Figure 3).

**Figure 4:**
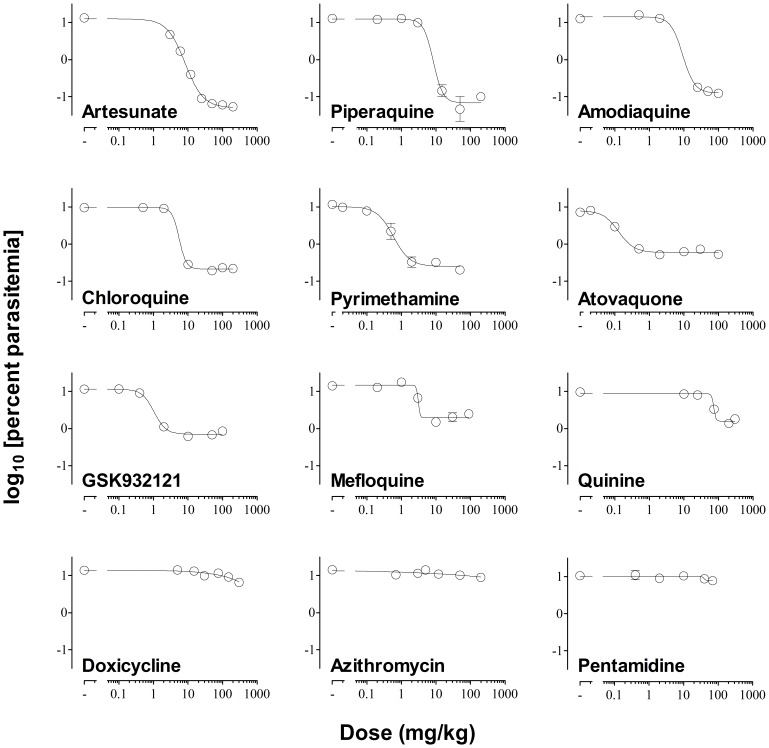
Best-fit dose–response curve. The plot shows the log_10_ [parasitemia at day 4] versus log_10_ [dose administered in mg/kg] of a set of antimalarial drugs used for validation of the *Plasmodium berghei* ED_90_-normalized in vivo assay. A minimum of five dose levels of each drug were used to fit the dose–response functions. The dotted line indicates the mean ED_90_ estimated for each drug.

**Figure 5:**
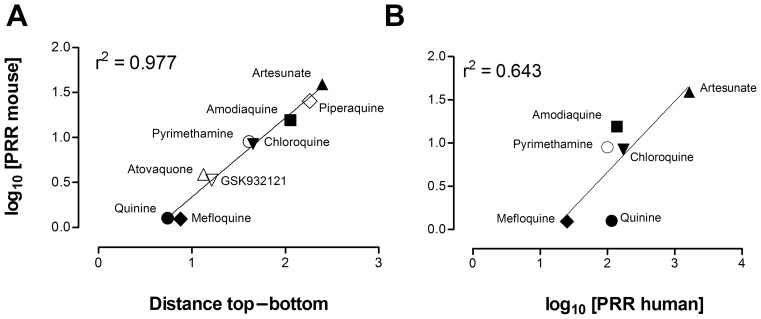
Analysis of parasite reduction ratio at 48 h (PRR_48h_). Evaluation of PRR_48h_ allowed validation of the *Plasmodium berghei* ED_90_-normalized in vivo assay in vehicle-treated control animals and against human data. (A) Correlation between the log_10_ [PRR_48h_] and the distance between top and bottom values of the logistic fit calculated in Figure 4 for each control antimalarial in CD1 mice infected with *P. berghei*. (B) Correlation of log_10_ [PRR_48h_] between CD1 mice infected with *P. berghei* in the screening assay format and humans infected with *P. falciparum*. Data on log_10_ [PRR_48h_] in humans are taken from [30]–[32].

The dose–response of the effective antimalarials used for assay validation showed marked differences in the distance between the top and the bottom of the logistic function fitted to each drug (Figure 4). The top–bottom distance correlated with the log_10_ [parasite reduction ratio at 48 h (i.e. PRR_48h_)] induced by each antimalarial (Figure 5A). However, the correlation between the log_10_ [PRR_48h_] of the efficacious control antimalarials in mice compared with humans was modest if quinine was considered (r^2^ = 0.64) (Figure 5B) [30]–[32], but was high if quinine was excluded (r^2^ = 0.84). These data suggest that the susceptibility of *P. berghei* and *P. falciparum* to quinine in vivo might be different. Moreover, the data support the contention that the rate of parasite clearance in vivo is significantly higher in humans than in mice. In conclusion, the *P. berghei* ED_90_-normalized assay detects differences in the PRR_48h_ of antimalarials in vivo by measuring parasitemia at day 4 and produces efficacy data in mice commensurable with that obtained in humans.

The sensitivity of the new in vivo screening assay may depend critically on the duration of treatment. In particular, the failure of doxicycline and azithromycin might indicate that the short duration of treatment would not detect compounds inducing parasite delayed death phenotypes [33]. To address this point we compared the potency (expressed as ED_90_) of each antimalarial compound in the Peters’ 4-day test and the ED_90_-normalized assay (Table 1). Both doxicycline and azithromycin were effective in the Peters’ 4-day test [34], [35], whereas pentamidine failed also in this experiment [29] (Table 1). Artesunate and piperaquine show similar potency in the Peters’ 4-day test and the ED_90_-normalized assay. For the other efficacious drugs, higher ED_90_ values were observed in the ED_90_-normalized assay versus the Peters’ 4-day test (Table 1). Interestingly, azithromycin (ED_90_ 156 mg/kg) showed detectable efficacy and mefloquine (ED_90_ 2 mg/kg) reached similar potency to that found in the Peters’ 4-day test when those compounds were administered for four consecutive days in an ED_90_-normalized assay. These results indicate that diminishing the duration of treatment in the ED_90_-normalized assay to two days *versus* four days in the Peters’ test, reduced the sensitivity for detecting compounds that provoke delayed death phenotypes.

**Table 1:** Comparison of the in vivo potency of a set antimalarial drugs in the ED_90_-normalized assay versus the Peters' 4-day test.

Compound	ED_90_-normalized assay	Peters' 4-day test
Atovaquone	0.4 (0.2–0.6)	0.06 (0.05–0.06)
Pyrimethamine	0.8 (1.2–1.6)	0.2 (0.1–0.4)
GSK932121	2.1 (1.0–3.0)	0.3 (0.26–0.4)
Mefloquine	4.2 (3.3–5.2)	2.0 (1.6–2.3)
Amodiaquine	5.1 (4–6.5)	3.2 (3.0–3.5)
Chloroquine	6.1 (3.9–7.5)	3.1 (2.8–3.5)
Artesunate	6.8 (6.0–7.5)	7.9 (7.4–8.3)
Piperaquine	7.8 (4.5–11.0)	4.2 (3.2–8.3)
Quinine	114.5 (78.7–166.3)	92.5 (81.3–110)
Pentamidine	>80.0	>40.0
Azithromycin	>200.0	13.0 (12.2–14.1)
Doxicycline	>300.0	170.8 (141.1–209.1)

Data are mean (95% CI).

### Identification of Leads Using an In Vivo Screening Assay

The feasibility of the in vivo screening approach to identify leads for drug development was addressed. The desired profile for an in vivo efficacious compound was an orally bioavailable compound when administered in aqueous vehicle (target product profile).

An in vivo screening protocol using the *P. berghei* ED_90_-normalized assay was evaluated by testing a set of compounds at a dose of 50 mg/kg suspended in water plus 1% methylcellulose. This level of potency was chosen as most currently available potent antimalarials have an ED_90_<15 mg/kg in the *P. berghei* ED_90_-normalized assay. All products were formulated at least 48–96 h before the first administration and identified with a correlative number. Compounds were administered to randomly selected mice on day three after infection. A compound was deemed effective in the assay if it reduced average parasitemia in treated mice by at least 40% with respect to vehicle-treated controls.

The screening conditions were not optimal for insoluble, unstable and slow acting compounds.Thus, a set of standard antimalarials of different solubility, chemical stability and mechanism of action were tested as internal controls of the screening conditions.

**Figure 6:**
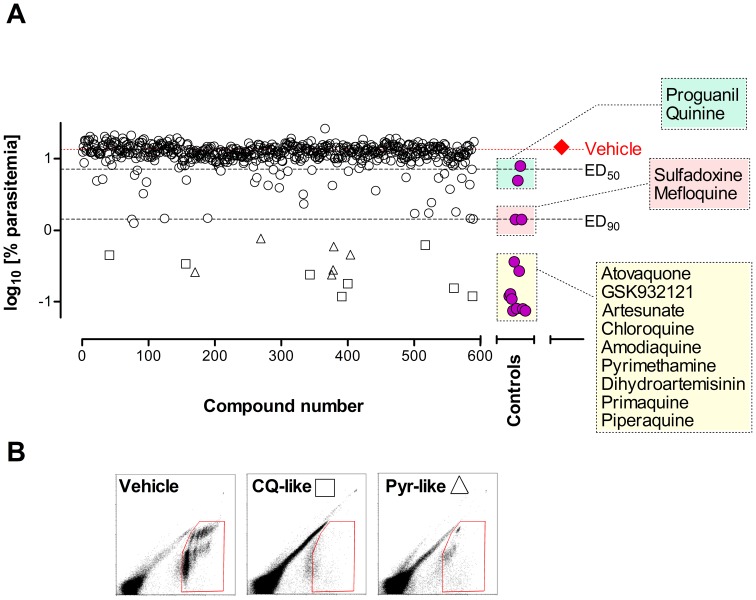
Screening of in vitro hits from TCAMS in the*Plasmodium berghei* ED_90_-normalized in vivo assay. (A) A collection of 590 compounds were screened at 50 mg/kg in 20% Captisol® given orally (open circles, open diamonds, open triangles). The series consisted of a first experiment of 50 compounds followed by 5 experiments of 100 compounds each and three additional experiments with 20, 7, and 13 compounds, respectively. Each experiment included a control group treated with vehicle (closed diamond) as a reference to calculate the percentage of inhibition of parasitemia in peripheral blood (dotted line). The response of standard antimalarials in the same assay is also presented (closed circles). Data shown are the mean log_10_ [parasitemia at day 4] of two mice per point. Open squares indicate compounds with YOYO-1_530/585_ flow cytometry patterns similar to chloroquine (CQ-like, potential fast killing compounds) whereas open triangles mark compounds with patterns similar to pyrimethamine (Pyr-like, potential non-fast killing compounds). (B) Patterns of YOYO-1_530/585_ flow cytometry method at day 4 for vehicle-, chloroquine- and, pyrimethamine-treated mice.

Figure 6A summarizes the evaluation of a sub-set of 590 compounds from the TCAMS collection having an IC_50_<2 µM, selected according to non-stringent standard criteria [36]. This TCAMS sub-set represents 4.4% of the 13,533 in vitro confirmed hits [13]. The compounds were evaluated only once in nine different experiments as soon as they were made available for formulation (10 mg of solid of each compound). All control antimalarials were found to be effective in the screening assay. Remarkably, most control compounds mapped to similar efficacy in the dose response curve shown in Figure 4. However, the results shown in Figure 6A indicate that the screening conditions may hamper the efficacy of compounds because compounds like dihydroartemisinin (insoluble and unstable) performed very well whereas proguanil (soluble and stable) was less efficacious than expected.

Compound availability was the rate-limiting step for the in vivo assay throughput because this assay could accommodate up to 500 compounds per month. Efficacy data were available one working day after the end of each in vivo assay. Thus, the *P. berghei* ED_90_-normalized assay is amenable for high-throughput in vivo evaluation of compounds.

The overall percentage of compounds in the TCAMS sub-set inhibiting more than 40% parasitemia *versus* vehicle-treated mice was 11.2% (66/590). The percentage of compounds that reduced the growth of *P. berghei* in vivo (ED_50_<50 mg/kg <ED_90_ equivalent to PRR_48h_ between 0.5 and 1) was 4.7% (28/590). Interestingly, 2.9% (17/590) of compounds from the TCAMS sub-set stopped parasite growth or induced rapid clearance of *P. berghei* from peripheral blood of mice in a way comparable to the most potent antimalarials currently available (ED_90_<50 mg/kg). These data indicate that using standard techniques of compound prioritization in the screening assay no less than 10% of the compounds tested are expected to be as efficacious as marketed antimalarials. Thus, an in vivo screening assay can provide advanced starting points for lead optimization.

The effects of drugs on the parasite population were analyzed to prioritize compounds for further development. Thus, a flow cytometry analysis was performed in mice treated with TCAMS compounds that showed significant efficacy in the in vivo screening. The analysis was performed using the same list-mode flow cytometry files used for measurement of the percentage of parasitemia. The patterns of light emission at 530 nm and 585 nm from parasites stained *ex vivo* with YOYO-1 allowed classification of compounds according to their similarity to standard antimalarials with different mechanisms of action (manuscript in preparation). Figure 6B shows that among the compounds that had ED_90_<50 mg/kg, the patterns of efficacy were compatible with chloroquine-like compounds (potential fast killing compounds) or pyrimethamine-like compounds (potential non-fast killing drugs). These data indicate that increasing the information content of the in vivo screening can help the prioritization of target product profiles early in drug discovery.

## Discussion

Our results support the contention that the use of in vivo screening early in drug discovery can accelerate the process until compounds reach clinical trials.

In vivo screening provides an integrated system in which drug efficacy can be assessed in a physiological context, i.e. encompassing host factors, drug disposition, and intrinsic drug anti-parasitic activity. Importantly, in vivo screening does not define *a priori* what a ‘good antimalarial’ should be in terms of disposition or in vitro activity. For example, artemisinin is very efficacious in vivo despite poor disposition in animals [37], and azithromycin is useful even though it has low antimalarial activity [38].

**Figure 7:**
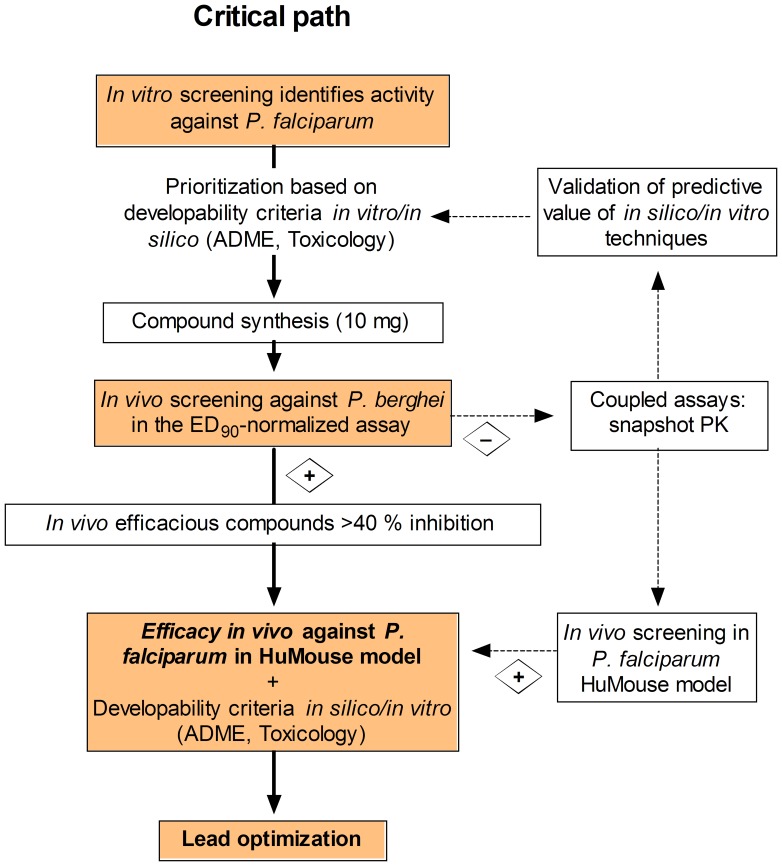
A new paradigm for the critical path of compound progression in antimalarial drug discovery. Compounds with activity in in vitro phenotypic assays are rigorously scored according to available in silico*/*in vitro absorption, distribution, metabolism and toxicity (ADMET) predictive techniques and theoretical chemical properties. Small amounts of compound (10 mg) are synthesized in order of priority for in vivo screening in the *Plasmodium berghei* ED_90_-normalized assay. Non-efficacious compounds showing significant exposure could be rescued for re-testing in the *P. falciparum* humanized mouse (HuMouse) model in order to discard the risk of species selectivity. Efficacious compounds are evaluated in vitro for drug metabolism/pharmacokinetics (DMPK), toxicity, and anti-parasitic activity for identification of development risks. The lead optimization program commences with full profiling of the lead compound: in vitro cytotoxicity and preliminary genotoxicity, in vivo DMPK (bioavailability and clearance in rodents), in vitro generation of resistance and killing rate activity, and in vivo dose–response efficacy in the *P. falciparum* HuMouse model. Further enrichment of the outcome of the *P. berghei* in vivo screening can be obtained by coupling high-content secondary endpoints to the screening, such as snapshot pharmacokinetic sampling, to provide valuable information for validation and refinement of ADMET predictive tools.

Usually, efficacy in vivo is the last step in the sequence of in silico and in vitro tests. During this process, iterative testing and synthesis of new derivatives in a compound series is performed to achieve an optimized ‘lead compound’ that has potent in vitro activity against *P. falciparum*, is not cytotoxic for a panel of mammalian cells in vitro (specific activity), is amenable for chemical modifications, shows no obvious predicted liabilities related to drug metabolism/pharmacokinetics or toxicity in humans (druggability), and is efficacious in animal models of malaria [39]. In this paper we propose a new paradigm, in which a high-throughput/high-content in vivo screening in a murine malaria model is the first step in lead identification (Figure 7). Accordingly, compounds active in vitro against *P. falciparum* and not overtly cytotoxic against the human hepatoma HepG2 cells [13] are tested against *P. berghei* in vivo. The compounds efficacious in the in vivo screening are orally bioavailable, not overtly toxic and potentially efficacious against different species of *Plasmodium*. These features represent a favorable starting point for optimization of the lead compound if, in fact, optimization is necessary.

Examples of the acceleration of the life cycle of drug discovery projects using the in vivo screening described in this paper are cyclopropyl carboxamides [40], [41], and indoline-containing derivatives of serotonin receptor 5-HT_2_ inhibitors [42]. Cyclopropyl carboxamides are compounds of a TCAMS series that were found to be very potent and rapidly parasiticidal in the *P. berghei* ED_90_-normalized in vivo screening assay. Despite their excellent pharmacological profile and outstanding efficacy against *P. falciparum* in vivo, the series showed a high propensity to generate resistance in vitro and was discontinued [41]. For cyclopropyl carboxamides, the full cycle until taking an informed no-go decision on the project was just six months. Similarly, the lack of outstanding efficacy in the *P. berghei* ED_90_-normalized in vivo screening assay contributed to the de-prioritization of further work on indolines that do not inhibit human serotonin receptor 5-HT_2_.

A key aspect of the ED_90_-normalized assay is that it is able to provide data that are meaningful to the treatment of human malaria. This was achieved by the extrapolation of human parasitological parameters to mice. The most useful parameter to model is the level of parasitemia in humans at the point of treatment initiation following hospital admission [28]. To translate parasitemia in patients to the corresponding parameter in mice (P_0_), parasitemia as a function of body weight was used. Body weight is appropriate for scaling many physiological constants among mammals [43], is almost equivalent to the difference of scale in blood volume between humans and mice (between 3–4 orders of magnitude), and has been used successfully in allometric pharmacokinetic/pharmacodynamic studies of antimalarials [44]. The calculated adult human-equivalent parasitemia in immunocompetent CD1 mice (∼1.5%) is easily detectable by flow cytometry and does not cause cerebral or severe anemia symptoms in mice.

The ED_90_-normalized assay was optimized to detect compounds that at least halted parasite growth in mice, i.e. parasitemia at the end of the assay ≤P_0_ (alternatively, PRR_48h_ ≥1). Moreover, compounds can be classified as a function of their capacity to induce the elimination of parasites in vivo or simply delay parasite growth. This is not surprising, because the PNSA design is based on human parameters of efficacy which are essentially linked to the rate at which parasites are cleared from peripheral blood [45]. In addition, the flow cytometry YOYO-1_530/585_ patterns can roughly inform on the mechanism of action of compounds tested in vivo. Taken together, this information might predict the expected pattern of efficacy in humans for a lead series at the very beginning of the drug discovery project. Furthermore, PNSA assays can be adjusted to accommodate different biological parameters to measure the effects of drugs on parasites, such as green fluorescent protein (GFP)-transformed parasites [46], [47], multi-parameter flow cytometry [48], [49] or protein or RNA arrays [50], [51]. The use of these technologies would also be useful for early characterization of the expected pattern of efficacy of new compounds in humans.

*Plasmodium berghei* is the species of choice for high-throughput in vivo screening because of its higher accessibility and widespread use in drug discovery [52]. Some specific genes of the rodent-adapted *P. berghei* may have significantly diverged from the human pathogen *P. falciparum* [53]. Thus, there is a risk of de-selecting *P. falciparum*-specific compounds. This is the case for pentamidine and other diamidine derivatives (DB289 and DB075), which are known to be efficacious against *P. falciparum* in humans but not against *P. berghei* [5], [29], [54]. The Pf-huMouse has also set up and validated in a PNSA assay format, though with a lower throughput compared with the *P. berghei* ED_90_-normalized screening assay [29], [55]. Figure 7 shows a schematic for integrating the two assays to streamline the selection of lead compounds. After failure in the *P. berghei* model, either because of low systemic exposure after oral administration or/and low specific activity against *P. berghei*, compounds could be diverted for evaluation in the Pf-huMouse model. Noteworthy, the critical path shown in Figure 7 is a tool that can be used in discovery programs for target product profiles (TPP) that require compounds with activity against the asexual erythrocyte stage of *Plasmodium* spp. [56].

The Pf-huMouse is the reference model for lead optimization and candidate selection [29], [55], [57]. At this stage of drug discovery, the evaluation of efficacy seeks to estimate the therapeutic index (TI). Essentially, this index is calculated as the difference between the levels of the drug in blood that are efficacious and those that are toxic. Our results suggest that the PNSA format may be more demanding for calculating TIs than other formats (e.g. Peters’ 4-day test) because the parameters of efficacy are more stringent (see Figure 1). Although this higher stringency could be regarded as an inconvenient, the PNSA format seems a more realistic approach for modeling treatments in humans. Actually, the measurements of efficacy in the PNSA format can be described by the homologous parasitological parameters of efficacy used in humans. Although this is one step forward in the validation of the Pf-huMouse, a full assessment of its validity as a predictive tool will require the development of a suitable metrics of efficacy [45] along with an understanding of the host- (physiology and drug disposition) and parasite-dependent (genetic background, cell cycle and susceptibility to drugs) variables [58].

The content of in vivo high-throughput screening could be boosted in a number of ways. Coupling pharmacokinetic analysis to the *P. berghei* efficacy screening would improve interpretation of efficacy results, so that only those compounds that had sufficient exposure, but which still failed against *P. berghei* would be reassessed in the Pf-huMouse model. Obtaining pharmacokinetic data would also allow analysis, refinement, and validation of the predictive power of in silico and in vitro ADMET techniques [29], [41], [59]. Validated in silico and in vitro ADMET techniques could be a powerful tool to prioritize compounds for in vivo screening and have the potential to improve the detection rate for efficacious compounds and the quality of leads [60], [61]. They should also be applicable to any drug discovery area [18].

Compound synthesis is the limiting factor to address the evaluation of the hits from malaria in vitro screenings (available as open access on EMBL-EBI at https://www.ebi.ac.uk/). Using the in vivo ED_90_-normalized screening assay described in this paper, around 10 mg of each compound would be needed. Meeting this synthetic challenge would require a global collaborative effort. This seems a feasible endeavor because a specialized laboratory could test approximately eight hundred compounds per month. Thus, a number of small coordinated laboratories could test the entire significant chemical diversity within the hits in less than two years. This is likely a pessimistic estimation of the time required to evaluate the hit set because the number of compounds that require testing could be significantly reduced by grouping compounds that share similar chemical structures (clustering).

According to our analysis, the timing of in vivo assays and the information obtained from them are the key parameters to accelerate drug discovery. Performing high-content in vivo assays at the hit level should allow predictions to be made regarding the expected compound profile in humans. This knowledge facilitates decision making on further investment according to the desired properties of the medicines. On the contrary, in vivo models aimed exclusively at increasing throughput may be misleading. For example, genetic alteration of parasites to facilitate their detection is not necessarily a substantial improvement for drug evaluation because it might compromise the susceptibility of the parasite to compounds of unknown properties [46], [47], [58].

In summary, the format of the *P. berghei* ED_90_-normalized assay required only two mice per experimental group and was optimized for the detection of compounds that prevent parasite growth or induce the elimination of *P. berghei* in vivo. The assay was shown to be a sensitive tool for detecting antimalarials that induce rapid clearance of *P. berghei* from mouse peripheral blood and excluded compounds with ‘delayed death’ phenotypes. Application of the assay to an in vivo screening campaign yielded a hit rate of around 11% from a sub-set of TCAMS that was selected according to standard non-stringent physicochemical criteria. About one third of the compounds deemed active in the screening assay were as efficacious in vivo as marketed antimalarial compounds.

These findings suggest that in vivo screening of the chemical diversity contained within the 20,000 antimalarial in vitro hits described in the literature is a feasible task. The only practical limitation is obtaining the required synthetic chemistry capacity in order to re-prepare compounds to support such an extensive screening exercise. Given the potential positive impact on malaria treatment and eradication, a multilateral collaborative project should be undertaken to meet this challenge.
